# Reference Ranges for the Clinical Laboratory Derived from a Rural Population in Kericho, Kenya

**DOI:** 10.1371/journal.pone.0003327

**Published:** 2008-10-03

**Authors:** Rukia S. Kibaya, Christian T. Bautista, Frederick K. Sawe, Douglas N. Shaffer, Warren B. Sateren, Paul T. Scott, Nelson L. Michael, Merlin L. Robb, Deborah L. Birx, Mark S. de Souza

**Affiliations:** 1 Walter Reed Project, U. S. Military HIV Research Program, Kericho, Kenya; 2 U. S. Military HIV Research Program, Henry M. Jackson Foundation, Rockville, Maryland, United States of America; 3 U. S. Military HIV Research Program, Rockville, Maryland, United States of America; 4 Centers for Disease Control and Prevention, Atlanta, Georgia, United States of America; 5 Department of Retrovirology, Henry M. Jackson Foundation, AFRIMS, Bangkok, Thailand; Instituto de Pesquisa Clinica Evandro Chagas, FIOCRUZ, Brazil

## Abstract

The conduct of Phase I/II HIV vaccine trials internationally necessitates the development of region-specific clinical reference ranges for trial enrolment and participant monitoring. A population based cohort of adults in Kericho, Kenya, a potential vaccine trial site, allowed development of clinical laboratory reference ranges. Lymphocyte immunophenotyping was performed on 1293 HIV seronegative study participants. Hematology and clinical chemistry were performed on up to 1541 cohort enrollees. The ratio of males to females was 1.9∶1. Means, medians and 95% reference ranges were calculated and compared with those from other nations. The median CD4+ T cell count for the group was 810 cells/µl. There were significant gender differences for both red and white blood cell parameters. Kenyan subjects had lower median hemoglobin concentrations (9.5 g/dL; range 6.7–11.1) and neutrophil counts (1850 cells/µl; range 914–4715) compared to North Americans. Kenyan clinical chemistry reference ranges were comparable to those from the USA, with the exception of the upper limits for bilirubin and blood urea nitrogen, which were 2.3-fold higher and 1.5-fold lower, respectively. This study is the first to assess clinical reference ranges for a highland community in Kenya and highlights the need to define clinical laboratory ranges from the national community not only for clinical research but also care and treatment.

## Introduction

Many human immunodeficiency virus (HIV-1) vaccine trials are slated for Phase I–III trials in Africa[1].The inception of the US President's Emergency Plan for AIDS Relief in 2004[2], with a mandate to treat 2 million HIV infections with anti-retroviral therapy (ART) by 2008 has accelerated the implementation of lymphocyte immunophenotyping in urban and rural areas in Africa as initiation of therapy is often predicated by absolute CD4+ T- lymphocyte counts. Central to any HIV vaccine and/or care and treatment program is the capability to measure absolute CD4 counts. CD4 counts are important in the context of breakthrough infections during HIV vaccine trials and informing treatment.

Phase I/II vaccine trials rely on the clinical laboratory for assessing safety, with particular emphasis on assays monitoring hematology, liver and kidney function. In addition, the treatment of HIV infection requires monitoring of drug toxicity on renal, hepatic and hematologic parameters. The majority of immunohematological and clinical chemistry reference ranges are based on North American or European data. Recently, there has been an increased initiative to determine immunohematologic reference ranges in Asia and Africa [3], [4], [5], [6], [7], [8], [9], [10], [11], [12], [13], [14], [15], [16]. Several factors including genetics, dietary patterns, sex, age and altitude can affect immunohematology and clinical chemistry reference ranges [17], [18]. Immunophenotyping of lymphocyte subsets has shown marked differences in CD4 T-cell counts depending on ethnicity. Lower CD4 T-cell numbers have been reported in Asians and Ethiopians compared to Caucasians [6], [11], [19], although absolute CD4 T cell counts in Africans from the Central African Republic have been reported to be similar to Europeans [10].

As early as 1941, hematology reference ranges were found to differ by race [20]. A study among four ethnic groups in the United Kingdom reported that black women had significantly lower white cell and neutrophil counts compared to Indian, Northern European and Oriental women [21]. Reference ranges for clinical chemistry, while well documented in North America [22], appear to have been little addressed in less industrialized countries. A reference range study for serum alanine aminotransferase (ALT) was conducted among Iranian blood donors and reported gender differences [23]. With the exception of a recent report by Saathoff et al., there are no published clinical chemistry reference range data for Africa [24]. The conduct of many Phase I/II HIV vaccine trials in Africa and Asia (http://www.iavireport.org/trialsdb/), and the increased global use of ART [25], [26], [27] support the need for national or regional reference ranges.

In addition to the need for local clinical reference ranges to successfully conduct HIV care and interpret data from HIV vaccine trials, these data guide clinical decision making for other medical issues. Our program has recently concluded a multi-national HIV vaccine trial in Kericho, Kenya, Mbeya, Tanzania and Kampala, Uganda. Prior to the execution of the vaccine trial in Kenya, there was an ongoing study to define the prevalence and incidence of HIV-1 infection in Kericho [28]. This allowed the opportunity to collect clinical laboratory reference ranges from this rural community. This report describes the collection and determination of reference ranges for a rural high-altitude population in Kenya.

## Methods

### Subjects

Study participants aged from 18 to 55 years were enrolled in a natural history cohort evaluating HIV-1 infection on a tea plantation in Kericho, Kenya [28], [29]. The predominant tribes in the cohort were Kalenjin, Kisii, Luo and Luhya [28]. Kericho is approximately 2042 m above sea level and lies 260 km northwest of Nairobi. Medical staff performed physical examinations and collected clinical histories. Participants who were not febrile, pregnant, HIV seropositive, and did not screen positive for syphilis and malaria were entered into the reference range sub-study. Values for 122 subjects were excluded from the analysis. No significant differences between this group and that included in the analyses were detected by either gender or age.

Reference value data were collected at study entry from 1,293 subjects: 848 (66%) males and 445 females from July to December 2003 for lymphocyte immunophenotyping. Reference values for clinical chemistry and hematology were collected from July to December 2004 from up to 1,541 individuals–1020 (66%) men and 521 women.

### HIV and Syphilis Serology

Serum samples were tested as described previously [30]. Briefly sera were initially screened for HIV antibodies using Genetic Systems rLAV EIA (BioRad Laboratories, Redmond, WA, USA). Reactive samples were tested in duplicate with the Vironostika HIV-1 Microelisa Systems (Organon Teknika, Durham, NC, USA) and repeatedly reactive samples confirmed with Genetic Systems HIV-1 Western blot (Bio-Rad Laboratories). Syphilis testing was performed using the Wampole Laboratories Impact RPR (Wampole Laboratories, Princeton, NJ, USA). Both the HIV and syphilis tests were approved by the US Food and Drug Administration (FDA).

### Malaria Testing

Thick film blood smears were prepared and read blinded by two independent readers. Discrepant results were resolved by a third reader. If the thick film was positive for parasites, thin films were prepared and parasites enumerated. A face-to-face questionnaire was also administered to each study participant to obtain a history of ever having had malaria infection and/or symptoms within the previous 6 months.

### Immunophenotyping

Blood was collected into a 7 ml Vacutainer tube containing EDTA (Becton Dickinson, Franklin Lakes, N.J., USA). Determination of lymphocyte subsets was performed using a FACSCalibur flow cytometer (Becton Dickinson Immunocytometry Systems, San Jose, CA, USA) and single platform technology. Two combinations of 4 monoclonal antibodies were used (anti-CD3/CD8/CD45/CD4 and anti-CD3/CD16+56/CD45/CD19) and TruCOUNT tubes (Becton Dickinson). Blood was stored at 20–25°C, stained within 48 h of collection and analyzed within 24 h of staining. Lymphocyte subsets were enumerated using MultiSET software (Becton Dickinson). Samples from 100 participants were excluded from the analysis due to the results failing our assay quality control criteria of the lymphocyte subset sum being outside of the 95%–105% range and/or the difference between replicate CD3 results being greater than 2%.

### Hematology

A complete blood count (CBC) and differential was performed on the identical blood sample used for immunophenotyping using an ACT 5Diff CP instrument (Beckman Coulter, Fullerton, CA, USA) within 24 h of specimen collection in accordance with manufacturer's instructions. The machine automatically dilutes a whole-blood sample of 45 µl in the CBC/Differential mode, lyses and counts.

### Clinical Chemistry

Samples for serum chemistry were collected in 3 ml serum tubes (Becton Dickinson). Serum was separated within 4 h of collection and analyzed within 12 h of blood draw using the Roche Cobas Integra 400 Chemistry Analyzer (Roche Diagnostics, Mannheim, Germany) for all analytes according to the manufacturer's instructions. Data from 22 subjects were totally excluded from the analysis due to one or more analyte result being clinically implausible.

### Quality Control

In addition to commercial controls run daily with each of the three instruments, the laboratory routinely participates (3 times per year) in external proficiency testing panels distributed by the College of American Pathologists for lymphocyte immunopehnotyping, hematology and clinical chemistry, and the United Kingdom National External Quality Assurance Service for lymphocyte typing. In the event that the daily commercial controls failed, testing was suspended.

### Ethics

Institutional Review Boards in the United States and Kenya approved the study. Written informed consent was obtained from each participant prior to blood collection.

### Statistical Analysis

All calculations for determining reference ranges were based on the guidelines found in the Clinical and Laboratory Standards Institute (CLSI: formerly National Council of Clinical Laboratory Services) guideline document C28-A2 [17]. Overall and gender-stratified data by analyte were evaluated for a normal distribution using the Shapiro-Wilks test, the Kolmogorov-Smirnov test with Lilliefors correction and the D'Agostino-Pearson test, and with diagnostic plots (i.e., boxplots and normal probability Q-Q plots). When the values of these tests were not statistically significant, we assumed that the distribution was Gaussian, and the 2.5% and 97.5% reference intervals were estimated using the mean and two standard deviations (S.D.). In the event of a non-Gaussian distribution, data were transformed (i.e., log-linear, the square root and inverse) to achieve normality, the mean and two S.D. were estimated and the data back-transformed to the original units. When normality could not be achieved, a non-parametric method was applied, which consists of ordering the data and estimating the sample 2.5 and 97.5 percentile to form the 95% reference intervals. Differences between genders were evaluated using the Student-*t* test or the Mann-Whitney U test. All statistical analyses were carried out using Stata v.9.0 (Stata Corporation, College Station, TX).

## Results

The mean age of all participants at study entry was 30 years. Men were marginally older than women, with mean ages of 31 years and 30 years, respectively. The frequency of malaria confirmed by blood film microscopy at baseline was 3%. Table 1 shows the median and 95^th^ percentile reference ranges for lymphocyte subsets for all 1293 eligible participants stratified by gender. Lymphocyte subset absolute numbers, with the exception of natural killer (NK) cells, were significantly different (P<0.05) between males and females, with women showing higher numbers for most subsets, with the exception of CD8+ T cell % and NK cell %. All study participants had a CD4 count of greater than 400 cells/µl, with 15% of men and 47% of women having a CD4 cell count exceeding 1000 cells/µl. The mean CD4 cell counts for men and women were 771 cells/µl and 1002 cells/µl, respectively, with an overall mean of 851 cells/µl.

**Table 1:** Lymphocyte subset reference ranges (median and 95th-percentile) derived from HIV-seronegative adults in Kericho, Kenya.

Parameter	Male	Female	All participants
	(N = 848)	(N = 445)	(N = 1293)
CD3+ T cells/µl^a^	1293 (711–2351)	1,660 (851–2742)	1415 (744–2634)
CD3+ T cells %^a^	72 (54–83)	74 (57–84)	73 (55–84)
CD4+ T cells/µl^a^	744 (407–1340)	982 (483–1651)	810 (421–1550)
CD4+ T cells %^a^	41 (30–55)	44 (32–56)	42 (30–55)
CD8+ T cells/µl^a^	454 (195–1046)	549 (225–1097)	486 (210–1081)
CD8+ T cells %	26 (14–40)	25 (15–38)	26 (14–39)
CD4∶CD8 ratio^a^	1.6 (0.8–3.3)	1.7 (0.9–3.3)	1.7 (0.9–3.3)
B-lymphocytes /µl^a^	218 (86–551)	295 (122–716)	244 (93–627)
B lymphocytes %^a^	12 (6–21)	13 (7–23)	12 (6–22)
NK cells /µl	252 (82–752)	255 (84–716)	253 (83–739)
NK cells %^a^	14 (5–30)	11 (4–27)	13 (4–30)

a : P value of <0.05 using the Wilcoxon rank test.

Table 2 shows selected hematology reference ranges for the Kericho cohort. There was a statistically significant difference (P<0.05) in all hematology parameters between men and women, with the former showing higher red blood cell counts, hemoglobin concentration and hematocrit levels compared to females. However women had higher white blood cell and platelet counts compared to men.

**Table 2:** Hematology reference ranges (median and 95th-percentile) derived from HIV-seronegative adults in Kericho, Kenya.

Parameter	Male	Female	All participants
	(N = 1020)	(N = 521)	(N = 1541)
Hemoglobin (g/dL) a	9.9 (8.3–11.3)	8.44 (5.9–10.0)	9.5 (6.7–11.1)
Hematocrit (%)^a^	47 (40–50)	40 (30–50)	45 (30–50)
Erythrocytes (10/^12^L) a	5.3 (4.4–6.3)	4.8 (3.7–5.6)	5.1 (4.0–6.2)
Platelets (10/^9^L) a	218 (115–366)	251 (124–444)	226 (120–411)
WBC (10^9^/liter) a	4.3 (2.7–7.5)	4.9 (3.0–9.1)	4.4 (2.8–8.2)
MCH (pg) a	30.1 (23.3–33.8)	28.9 (21.3–33.0)	29.8 (22.4–33.5)
MCV (fl) a	88.0 (71.4–98.2)	84.9 (66.0–95.7)	87.0 (68.8–97.2)
MCHC (g/L) a	342 (324–353)	339 (322–352)	341 (322–353)
Neutrophils (10/^9^L) a	1780 (871–4324)	1960 (987–5558)	1850 (914–4715)
Neutrophils (%)	42 (20–70)	42 (20–70)	42 (20–60)
Lymphocytes (10/^9^L) a	1860 (1120–3160)	2160 (1290–3957)	1950 (1140–3454)
Lymphocytes (%)	45 (20–60)	45 (20–60)	45 (20–60)
Monocytes (10/^9^L) a	280 (130–585)	300 (160–640)	290 (130–600)
Monocytes (%)^a^	7 (3–12)	6 (3–11)	7 (3–11)
Eosinophils (10/^9^L) a	150 (30–1080)	170 (34–1218)	160 (30–1139)
Eosinophils (%)	4 (1–20)	4 (1–20)	4 (1–20)
Basophils (10/^9^L)	30 (10–90)	30 (10–70)	30 (10–80)
Basophils (%)^a^	0 (0–2)	0 (0–1)	0 (0–2)

a : P value of <0.05 using the Wilcoxon rank test.

Clinical chemistry values for the Kericho cohort are shown in Table 3. The full range of analytes was not run for all subjects, primarily due to insufficient test kits for that analyte, or in rare cases, failure of the commercial quality control materials. Except for sodium and glucose, all parameters were significantly different between genders, with higher median values in men for most analytes (12/17). Comparing the reference ranges for hepatic and renal function shows that males had elevated liver enzymes and creatinine levels compared to females, but these were not clinically significant.

**Table 3:** Clinical Chemistry reference ranges (median and 95th-percentile) derived from HIV-seronegative adults in Kericho, Kenya.

ANALYTE	N	Male	N	Female	N	All Participants
**Enzymes**						
ALT (U/L) a	1020	22.3 (10.8–53.9)	521	16.8 (8.6–47.0)	1541	20.2 (9.6–52.0)
AST (U/L) a	1016	23.9 (14.9–45.3)	517	19.1 (13.1–38.1)	1533	22.2 (13.8–42.3)
Amylase (U/L) a	792	85.1 (40.0–171.3)	413	78.6 (36.0–147.8)	1205	82.4 (38.3–163.0)
Lactate dehydrogenase (U/L) a	820	174 (124–259)	420	181 (131.5–295.4)	1240	176 (126.0–263.9)
**Serum Proteins**						
Albumin (g/L) a	1020	42.9 (36.9–48.5)	521	41.7 (34.4–47.5)	1541	42.5 (35.8–48.1)
**Metabolism**						
Bilirubin, total (µmol/L) a	1020	12.2 (5.6–41.9)	521	9.6 (4.4–26.8)	1541	11.2 (4.9–39.9)
Bilirubin, direct (µmol/l) a	1020	3.1 (1.3–9.5)	521	2.4 (0.8–6.7)	1541	2.8 (1.1–8.8)
Glucose (mmol/L)	1020	4.1 (3.0–5.6)	521	4.0 (3.2–5.7)	1541	4.1 (3.1–5.7)
Cholesterol (mmol/L) a	996	3.8 (2.5–5.5)	512	3.9 (2.6–5.9)	1508	3.8 (2.6–5.7)
Triglycerides (mmol/L) a	974	0.9 (0.4–2.7)	503	0.8 (0.4–2.5)	1477	0.9 (0.4–2.6)
**Kidney Function**						
Creatinine (µmol/L) a	1019	77 (62–106)	521	66 (51–91)	1540	74 (55–102)
Urea nitrogen (mmol/L) a	1020	2.8 (1.5–4.6)	521	2.5 (1.4–4.6)	1541	2.7 (1.4–4.6)
**Electrolytes**						
Sodium (mmol/L)	1020	146.5 (141.8–152.1)	521	146.5 (140.3–155.3)	1541	146.5 (141.4–152.5)
Potassium (mmol/L) a	1017	4.6 (3.9–5.8)	518	4.5 (3.8–5.8)	1535	4.6 (3.9–5.8)
Chloride (mmol/L) a	1020	105.0 (100.4–110.8)	521	106.9 (101.1–113.4)	1541	105.6 (100.5–111.7)
Carbon dioxide (mmol/L) a	1020	23.5 (18.9–29.0)	521	21.6 (16.8–26.9)	1541	22.9 (17.7–28.5)

a : P value of <0.05 using the Wilcoxon rank test.

Comparing the immunophenotyping data with the US reference range data provided by Becton-Dickinson showed good agreement with the North American population although the upper limit of the reference range for all T cell absolute counts was higher in the North American population (Table 4). The reference range for the US population was also wider for all lymphocyte subsets, except NK cells, although the US sample size was considerably smaller (N = 164), which may account for the wider range. The reference ranges for CD4 T cell numbers and percentages for the Kenyan group were higher than those reported from Tanzania and Ethiopia [11], [24]. The CD4∶CD8 reference range ratio was very similar for the Kenyan and Tanzanian participants, but the lower limit was at least 2-fold higher than that seen for Ethiopians [11], [24].

**Table 4:** Comparison of Lymphocyte Reference Ranges from Kericho, Kenya with those from other countries.

Parameter	Kericho	Tanzania[24]	Ethiopia[11]	USA^a^
CD3+ T cells/µl	744–2634	683–2106	854–2556	723–2737
CD3+ T cells %	55–84	52–80	NA	56–86
CD4+ T cells/µl	421–1550	406–1392	366–1235	404–1612
CD4+ T cells %	30–55	27–52	NA	33–58
CD8+ T cells/µl	210–1081	188–990	311–1618	220–1129
CD8+ T cells %	14–39	13–40	NA	13–39
CD4∶CD8 ratio	0.9–3.3	0.8–3.2	0.4–2.4	NA
B-lymphocytes /µl	93–627	109–637	51–419	80–616
B lymphocytes %	6–22	6–24	NA	5–22
NK cells /µl	83–739	123–801	75–581	84–724
NK cells %	4–30	6–34	NA	5–26

a : Reference ranges provided by Becton-Dickinson with the MultiTEST IMK Kit Reagent package (12/2000;23-3602-02).

b :NA-Not Available.

The Kenyan hematology parameters showed marked differences for hemoglobin, leukocyte counts and eosinophils compared with a North American group (Table 5) [22]. The lower and upper reference ranges for hemoglobin and eosinophils, respectively, were 2 times lower for the former in women and 2.5 times higher for the latter compared to the US group [22].

**Table 5:** Hematology reference ranges from Kericho, Kenya compared to other sources in Africa and the United States of America.

Parameter	Kenya	Tanzania[24]	Ethiopia[11]	USA[22]
Hemoglobin (g/dL)				
All	6.7–11.1	11.7–17.2	NA^a^	NA
Male	8.3–11.3	13.7–17.7	13.9–18.3	13.5–17.5
Female	5.9–10.0	11.1–15.7	12.2–16.6	12.0–16.0
Hematocrit %				
All	35.0–55.0	36.0–53.0	NA	NA
Male	40.0–50.0	40.2–53.7	41.6–55.1	41.0–53.0
Female	30.0–50.0	36.2–46.8	35.3–48.8	36.0–46.0
RBC (×10^12^/L)				
All	4.0–6.2	4.0–6.1	NA	NA
Male	4.4–6.3	4.4–6.3	4.3–5.9	4.5–5.9
Female	3.7–5.6	3.8–5.6	3.7–5.2	4.0–5.2
Platelets (×10^9^/L)	120–411	150–395	NA	150–350
WBC (×1,000)	2.8–8.2	3.0–7.9	3.0–10.2	4.5–11.0
MCH (pg)	22.4–33.5	23.6–33.1	NA	26.0–34.0
MCV (fl)	68.8–97.2	77.6–98.1	NA	80.0–100.0
MCHC (g/dl)	322–353	306–349	NA	310–370
Neutrophils (×10^9^/L)	0.9–4.7	1.1–4.7	NA	NA
Neutrophils %	20–60	32–69	31–78	40–70
Lymphocytes (×10^9^/L)	1.1–3.5	1.1–3.0	NA	NA
Lymphocytes %	20–60	21–57	17–59	22–44
Monocytes (×10^9^/L)	0.1–0.6	NA	NA	NA
Monocytes %	3–11	NA	3–10	4–11
Eosinophils (×10^9^/L)	0.03–1.1	NA	NA	NA
Eosinophils %	1–20	NA	NA	0–8
Abs Basophils (×10^9^/L)	0.01–0.08	NA	NA	NA
Basophils %	0–2	NA	NA	0–3

a : NA-Not Available.

The Kenyan reference ranges for clinical chemistry analytes showed higher enzyme and electrolyte levels compared to North Americans [22]. The Kericho data are more comparable to the Mbeya, Tanzania data [24] than the US reference ranges (Table 6). The reference range for blood urea nitrogen was much lower than that for the US group, with the upper limit for the latter being 1.5-fold more than that of Kericho.

**Table 6:** Chemistry Reference Ranges for adults from Kericho, Kenya compared to those from two other countries.

ANALYTE	Kenya	Tanzania[24]	USA[22]
**Enzymes**			
ALT (U/L)	0^a^–52	0–48	0–35
AST (U/L)	0–42	0–48	0–35
Amylase (U/L)	38–163	43–164	60–180
Lactate dehydrogenase (U/L)	126–264	127–264	100–190
**Serum Proteins**			
Albumin (g/L)	36–48	36–50	35–55
**Metabolism**			
Bilirubin, total (µmol/L)	4.9–39.9	5.2–41.0	5.1–17.0
Bilirubin, direct (µmol/l)	1.1–8.8	0.7–8.2	1.7–5.1
Glucose (mmol/L)	3.1–5.7^b^	2.9–5.2 b	4.2–6.4 c
Cholesterol (mmol/L)	0–5.7	0–5.5	0–6.2
Triglycerides (mmol/L)	0–2.6	0–2.9	0–1.8
**Kidney Function**			
Creatinine (µmol/L)	0–102	0–90	0–133
Urea nitrogen (mmol/L)	1.4–4.6	1.5–5.0	3.6–7.1
**Electrolytes**			
Sodium (mmol/L)	141–152	134–142	136–145
Potassium (mmol/L)	3.9–5.8	3.8–5.5	3.5–5.0
Chloride (mmol/L)	100–112	98–107	98–106
Carbon dioxide (mmol/L)	18–28	19–30	21–30

a : 2.5^th^ percentile set to zero to match comparison ranges, and thus differs from Table 3.

b : Non-fasting measurement.

c : Fasting measurement.

## Discussion

Our data collection method was in accordance with the rigorous CLSI guidelines for determining laboratory reference ranges, which recommends a minimum of 153 subjects for a 95^th^ percentile clinical reference range determination with 95% confidence [17].

The Kenyan group CD4 reference range is also comparable to that measured in Nigeria (309–1327 cells/µl) [3] and a study in Dar es Salaam (405–1500 cells/µl) [15], despite differences in assay methodologies. The study conducted in Mbeya, Tanzania was performed in a similar rural population and identical methodology to our study but showed a narrower and lower reference range (406–1392 cells/µl) [24]. As seen with most previous studies, the CD4 median absolute count was higher in women compared to men [3], [5], [8], [15], [16], [24], [31]. The CD4 reference range in Kenyan women is comparable to that of African-American women in the USA. A recent study of 515 African-American women using two-color flow cytometry reported a mean CD4 cell count of 1055 cells/µl [32].

The hematology data showed gender differences for most parameters, and the trend agreed with most published studies [8], [10], [24]. The finding of higher values for hemoglobin, hematocrit and erythrocytes in males compared to females may be partly due to the influence of androgen on erythropoiesis, menstrual loss and parity. Compared to other African sites, such as Akaki, a high-altitude (2100 m) site in Ethiopia, and Mbeya, Tanzania, our reference ranges were similar, with the exception of hemoglobin, which was much lower in the Kenyan group [11], [24]. One major difference in our rural population compared to those from industrialized nations was the higher eosinophil count. Eosinophilia may be related to parasitemia, as our medical examination did not include intestinal helminthes, which is a limitation of this study. A stool parasitologic survey performed in 2002 within 100 km of Kericho, reported a prevalence of *Ascaris lumbricoides*, hookworm and *Trichuris trichuri* of 10, 4 and 0.1%, respectively, but no other parasites were assessed [33].

The two surprising findings in this study were the reference ranges for hemoglobin and the differential for neutrophils. It could be argued that the difference between our hemoglobin data and that from Ethiopia might be due to diet and methodology. The major food of the people of the Akaki region has a very high iron content [11]. Additionally, Kericho is about 400 m–600 m higher than the Tanzanian study site, therefore, the hemoglobin levels should be higher than Mbeya [34]. Interestingly, while iron was not directly measured in this cohort, 26% of women and 9% of men had an MCV of <80 fl, which is an indirect marker of iron deficiency. The difference in the neutrophil % reference range for this study and that from Mbeya may be the result of differences in pathogen exposure and/or population genetics, or diet. It has been argued that benign ethnic neutropenia should be considered a variation of normal as it does not appear to incur a clinical disadvantage [35].

White blood cell and platelet counts and hemoglobin levels are used as inclusion and/or exclusion criteria in Phase I/II vaccine trials. The Division of AIDS (DAIDS) has hematological criteria for grading the severity of potential vaccine related adverse events (AE): http://www3.niaid.nih.gov/research/resources/DMIDClinRsrch/toxtables.htm. The lower limit of the Kenyan group's reference range neutrophil count would be classified as a moderate adverse event (range 750–999×10^9^ /L) and the lower limit of the hemoglobin reference range for the group would have been graded as a severe adverse event (range 6.5–7.4 g/dL). However, stratifying hemoglobin by gender shows that the lower limit for males would have been graded as a moderate AE, but that for women would have been classified as potentially life-threatening. A hematologic reference range study conducted in a community living at a lower altitude (1175 m) in Uganda, using identical instrumentation to our study, reported a lower reference range for hemoglobin of 11.1 g/dL and 10.1 g/dL for men and women respectively, based on the lower 5^th^ percentile and not the 2.5^th^ percentile of the current study [8]. It is difficult to establish why our reference range values for hemoglobin were so low compared to other African sites using identical methodology. It is unlikely to be due to malaria being under-diagnosed, as the study team was proficient in malaria diagnostics by blood smear. One report from a nearby highland community in Kenya reported a prevalence of *Plasmodium falciparum* of 5%, which was marginally higher than our study [33]. The lower reference range limit for platelet count in this study would have been a mild adverse event (range 100–125×10^9^ /L) using the DAIDS AE tables. Sickle cell anemia and malnutrition may have also played a role in the low values for hemoglobin. However a sickle cell trait survey conducted in the region demonstrated a frequency of 2.5% in tribes representing 52% of the group [28], [36]

The clinical chemistry reference range data from Kericho compared to the US are similar for most parameters, with the exception of urea nitrogen, which was considerably lower in the Kenyan study. Interestingly, urea nitrogen for the Tanzanian study using the identical methodology was in better agreement with this study than the US data [22]. Similarly, the upper reference value for total bilirubin in the Kenyan group, as seen with the Tanzanian study, was over 2-fold that of the US data [22], [24]. The glucose measurement in the current study was for non-fasting glucose, unlike the US data [22], and the lower limit of the reference range would have been considered a mild serious adverse event by the DAIDS toxicity tables (range: 3.1–3.6 mmol/L) It should be stressed that neither the methodology nor the sample size used for the US reference range data is known.

This study provides the first lymphocyte, hematology and clinical chemsitry reference range data for Kericho, Kenya, and to date is the largest cohort from which reference range data has been collected in Africa. While our data showed similarities with industrialized countries, there were striking differences, particularly for hematology and clinical chemistry. WHO has designated hemoglobin and glucose measurement as essential laboratory services for sub-Saharan Africa [37]. The reference range data generated from the Kericho study showed that these two parameters, particularly the former, are widely different to those of the US. Using recent data from the US which defines anemia in African-American men and women as hemoglobin <12.9 g/dL and <11.5 g/dL, respectively, the entire Kericho study population would have been classified as anemic [38]. Our study has shown that strict adherence to reference ranges developed in industrialized countries for HIV vaccine and/or treatment trials could exclude many healthy Kenyans from participating in these studies. Similarly, the use of “Western” reference ranges for clinical management could lead to unnecessary treatment. Our study not only allowed definition of reference ranges for a rural area in Kenya, but also enhanced local laboratory capacity as it was conducted under Good Clinical Laboratory Practices guidelines [39] thus preparing the site for clinical trials with potential product licensure by the US FDA.
